# Supplementary material for: The phosphorylation of carboxyl-terminal eIF2α by SPA kinases contributes to enhanced translation efficiency during photomorphogenesis
Source: Nat Commun. 2024 Apr 24;15:3467. doi: 10.1038/s41467-024-47848-7 (PMC11043401; doi:10.1038/s41467-024-47848-7)
Supplement: Supplementary file 1 — Supplementary Information [file 41467_2024_47848_MOESM1_ESM.pdf]

**FIGURE LEGENDS:**

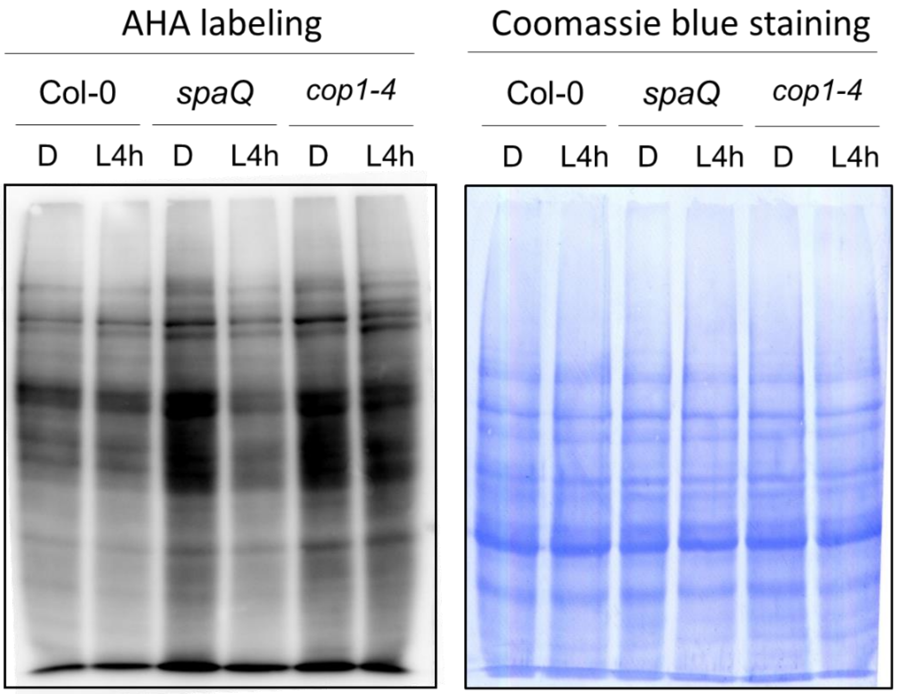


| | Col-0 | *spaQ* | *cop1-4* |
|---|---|---|---|
| Dark (D) | 1.00 | 1.46 ± 0.24 | 1.33 ± 0.16 |
| Light (L) | 1.22 ± 0.06 | 0.98 ± 0.09 | 1.44 ± 0.07 |

**Supplementary Figure 1: *spaQ* and *cop1-4* mutants exhibited enhanced new protein synthesis in the dark but repressed new protein synthesis upon light exposure.**

The figures showed *de novo* protein synthesis (left) examined by immunoblotting using antibodies against streptavidin, and the total protein content (right) stained with Coomassie blue. Four-day-old etiolated seedlings of Col-0, *spaQ* and *cop1-4* were incubated either under dark (D) or illuminated under white light for 4 h (L4h) before sampling. The table below the figures indicated the relative signal intensities of *de novo* protein synthesis normalized to total protein content. The ratio of the dark condition of Col-0 was set to 1 for analysis. Data of signal intensities are mean ± SD from three biological replicates.

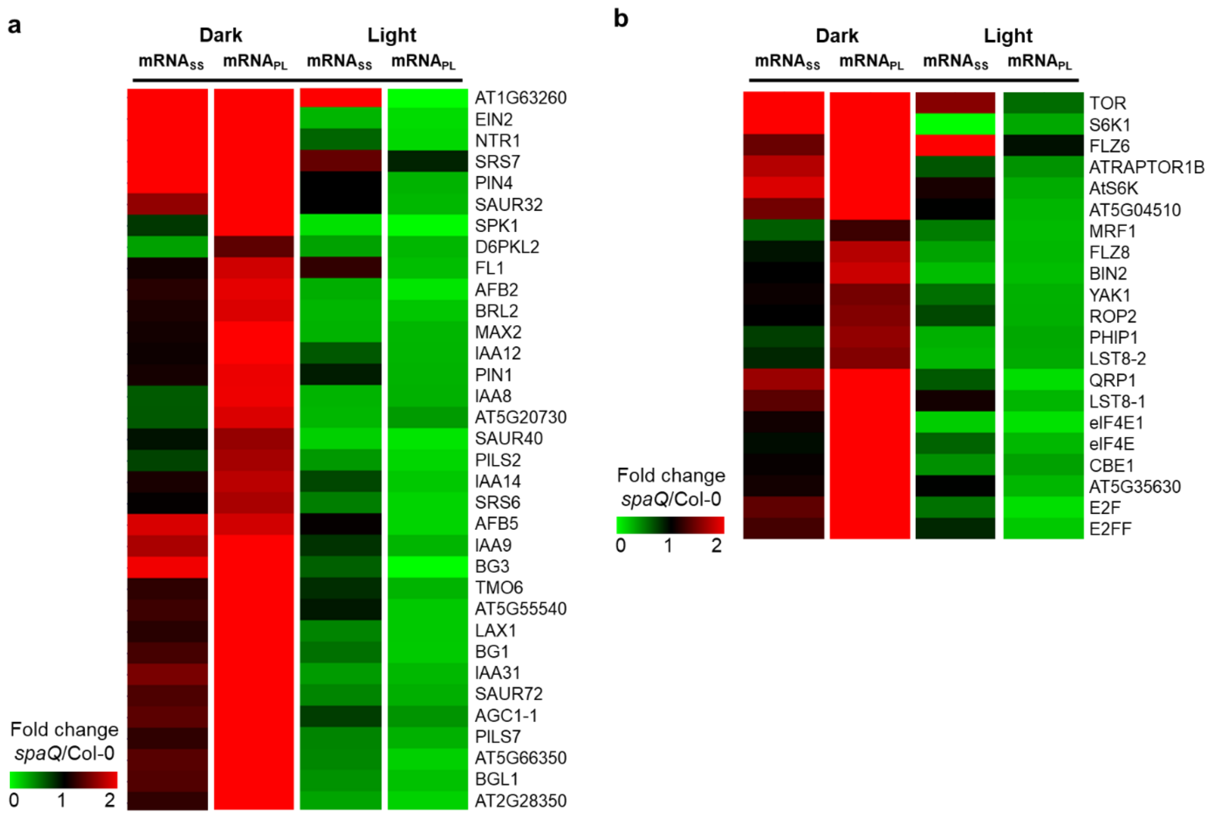


**Supplementary Figure 2: SPAs promote the expressions of genes involved in auxin signal transduction and TOR pathway at the translational level upon light exposure.**

**a, b** Heat maps showing the expressions in fold change of SPA-regulated genes involved in auxin signal transduction (a) and TOR pathway (b). Four-day old Col-0 and *spaQ* etiolated seedlings incubated either under dark or illuminated 4 h white light were used as the samples. Steady-state mRNAs ($mRNA_{SS}$) and polysome-bound ($mRNA_{PL}$) were isolated in parallel. Extreme red and green colors indicate ratio 2 and ratio 0 of the fold change, respectively.

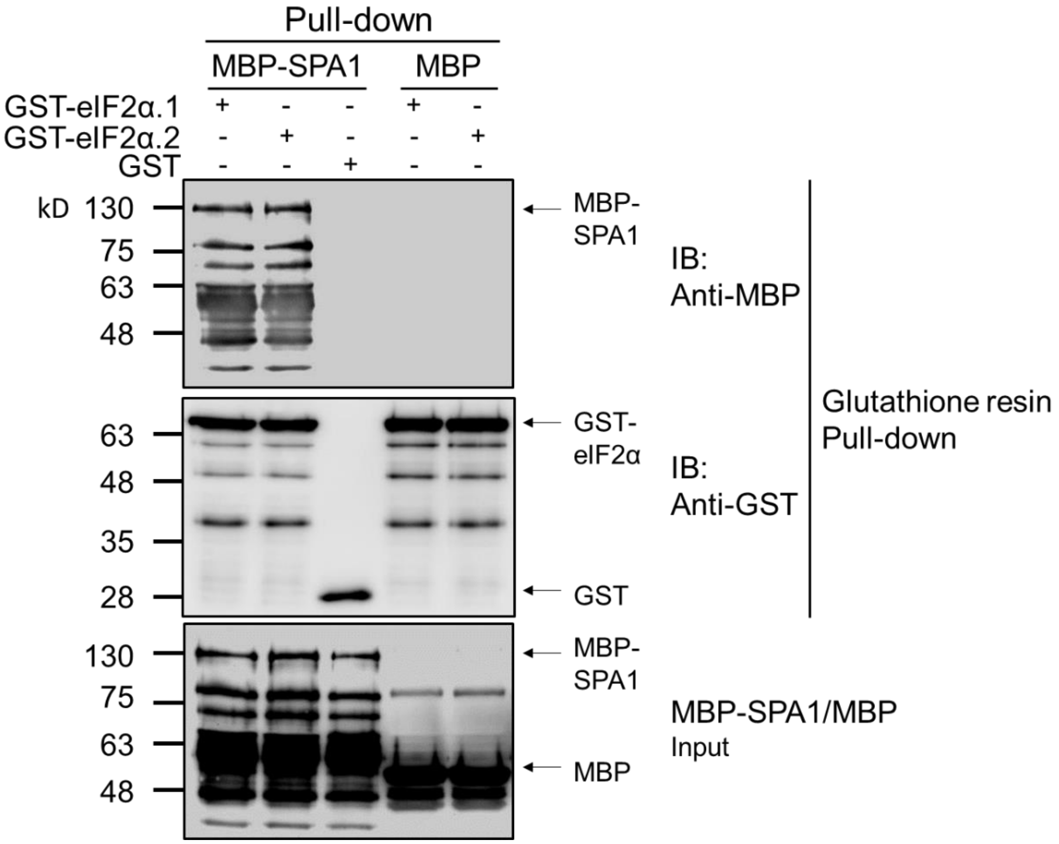


**Supplementary Figure 3: SPA1 interacts with both eIF2α.1 and eIF2α.2 *in vitro*.**

*In vitro* pull-down assay shows that eIF2α interacts with SPA1. The GST-eIF2α, GST, MBP-SPA1 and MBP were expressed and extracted from *E. coli*. Three biological repeats of data showed the same results. The tag proteins, GST and MBP, were used as a negative control. The proteins were examined by immunoblotting using antibodies against MBP and GST.

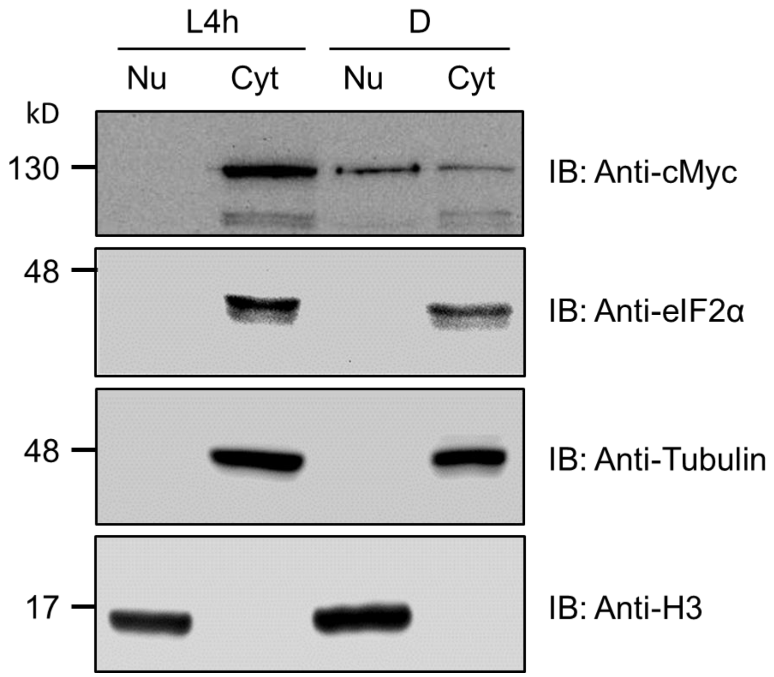


**Supplementary Figure 4: Detection of SPA1 proteins in the cytosolic and nuclear fractions.** The amount of TAP-SPA1 and eIF2α levels in the nuclear (Nu) or cytosolic fraction (Cyt) in 4-day-old TAP-SPA1 plants incubated either under dark (D) or illuminated under white light for 4 h (L4h) before sampling. The equal amount of sample was extracted for cytosolic protein. The equal amount of buffer was added to resuspend the pellets containing nuclear proteins and 1/2 of the resuspended nuclear proteins were loaded. Three biological repeats of data showed the same results. Anti-tubulin and anti-histone H3 antibodies were used to detect marker proteins for cytosolic and nuclear fractions, respectively.

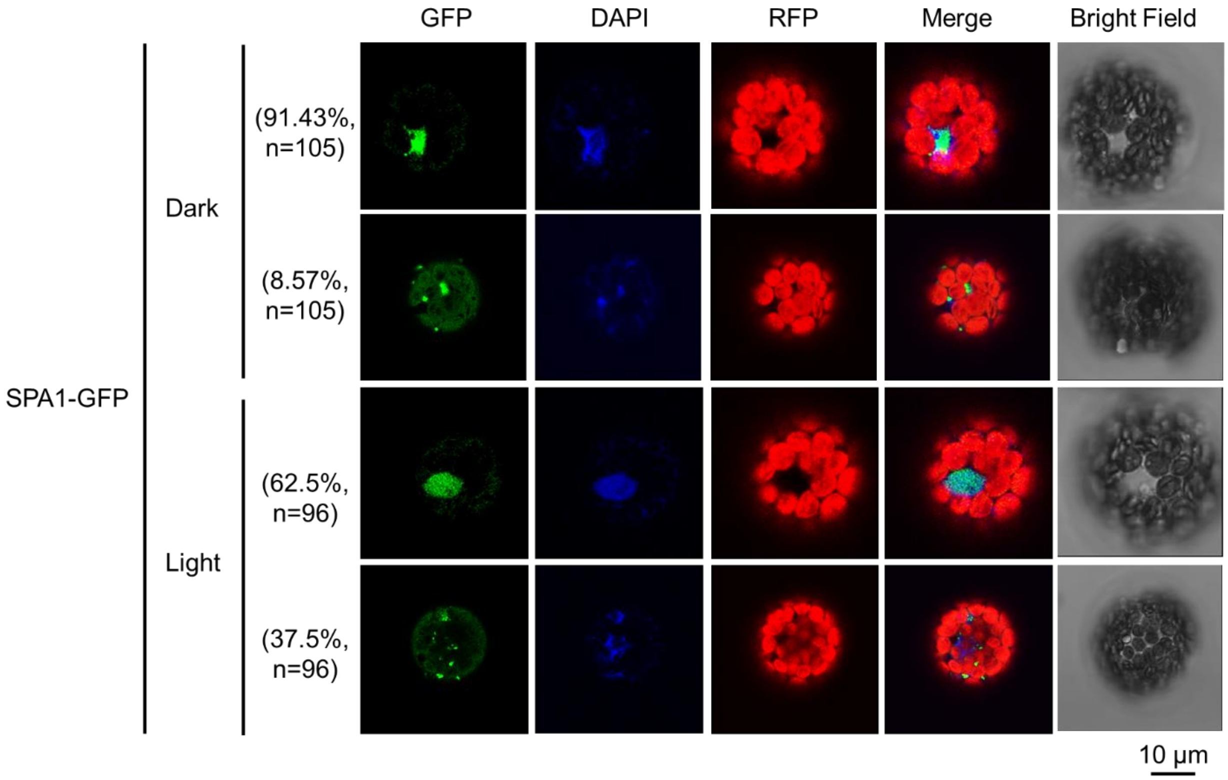


**Supplementary Figure 5: Subcellular localization of SPA1-GFP under dark and light conditions.**

Full-length SPA1 was fused with green fluorescence protein (GFP) and transiently expressed in *Arabidopsis* protoplasts. The dark-treated protoplasts (Dark) were incubated under dark for 2 h before observation, whereas the light-treated protoplasts (Light) were incubated under light for 2 h before observation. In the Dark sample, 105 successfully transformed protoplasts were analyzed for SPA1 localization, and 96 protoplasts were analyzed in the Light sample. The GFP, Chl, 4', 6'-diaminophenylindole (DAPI; nucleus staining), red fluorescence protein (RFP; chlorophyll fluorescence), merge (merged image of YFP, DAPI and RFP) and bright field were shown for each type of transformation combination. Bar=10 μm.

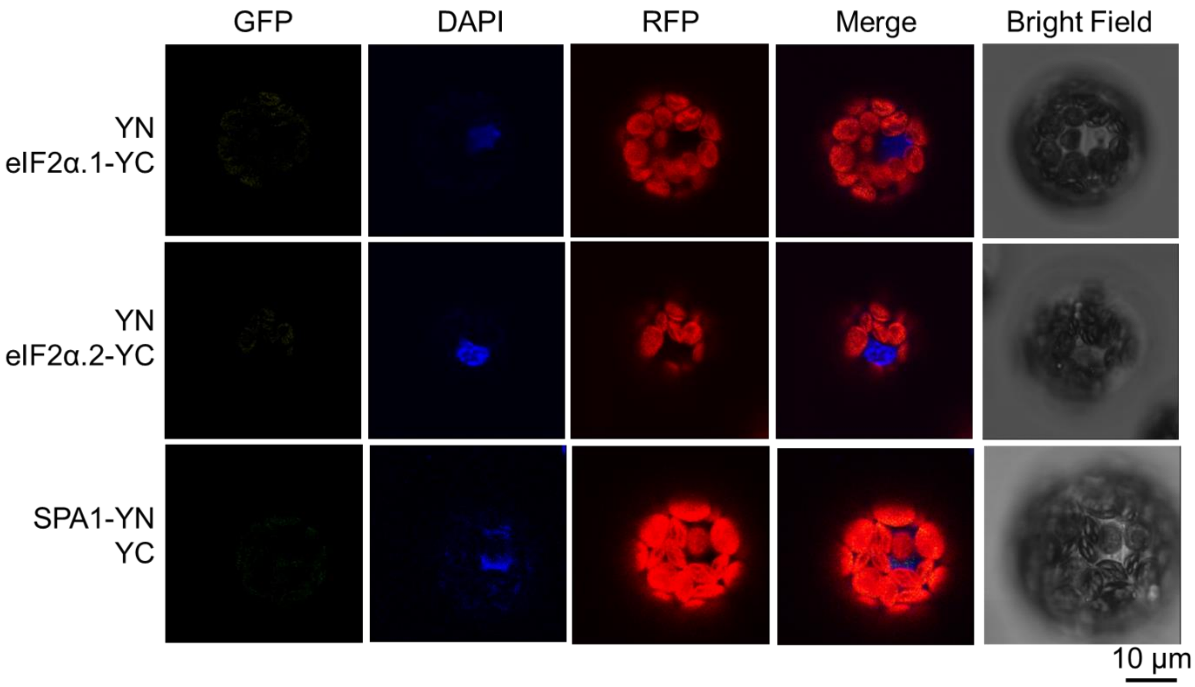


**Supplementary Figure 6: The negative controls for BiFC assay did not exhibit any signals.** Full-length SPA1 and eIF2α.1/eIF2α.2 were fused with N- and C-terminal yellow fluoresce protein (YN and YC), respectively. All of the transformed protoplasts were incubated under light for 2 h before observation. Empty YN and YC vectors were used as negative controls. The YFP, Chl, 4', 6'-diaminophenylindole (DAPI; nucleus staining), red fluorescence protein (RFP; chlorophyll fluorescence), merge (merged image of YFP, DAPI and RFP) and bright field were shown for each type of transformation combination. Bar=10 μm.

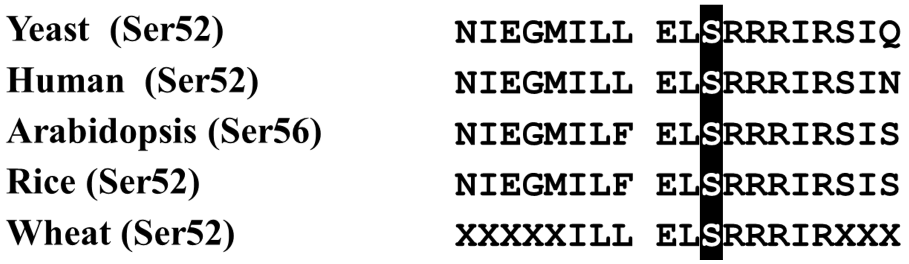


**Supplementary Figure 7: Alignment of amino acids surrounding the phosphorylation site at the N-terminal end of eIF2α showing conservation of the sequence in different species.** (adapted from Zhang et al., 2008)

The species include plants, animals and fungi. The target serine is highlighted.

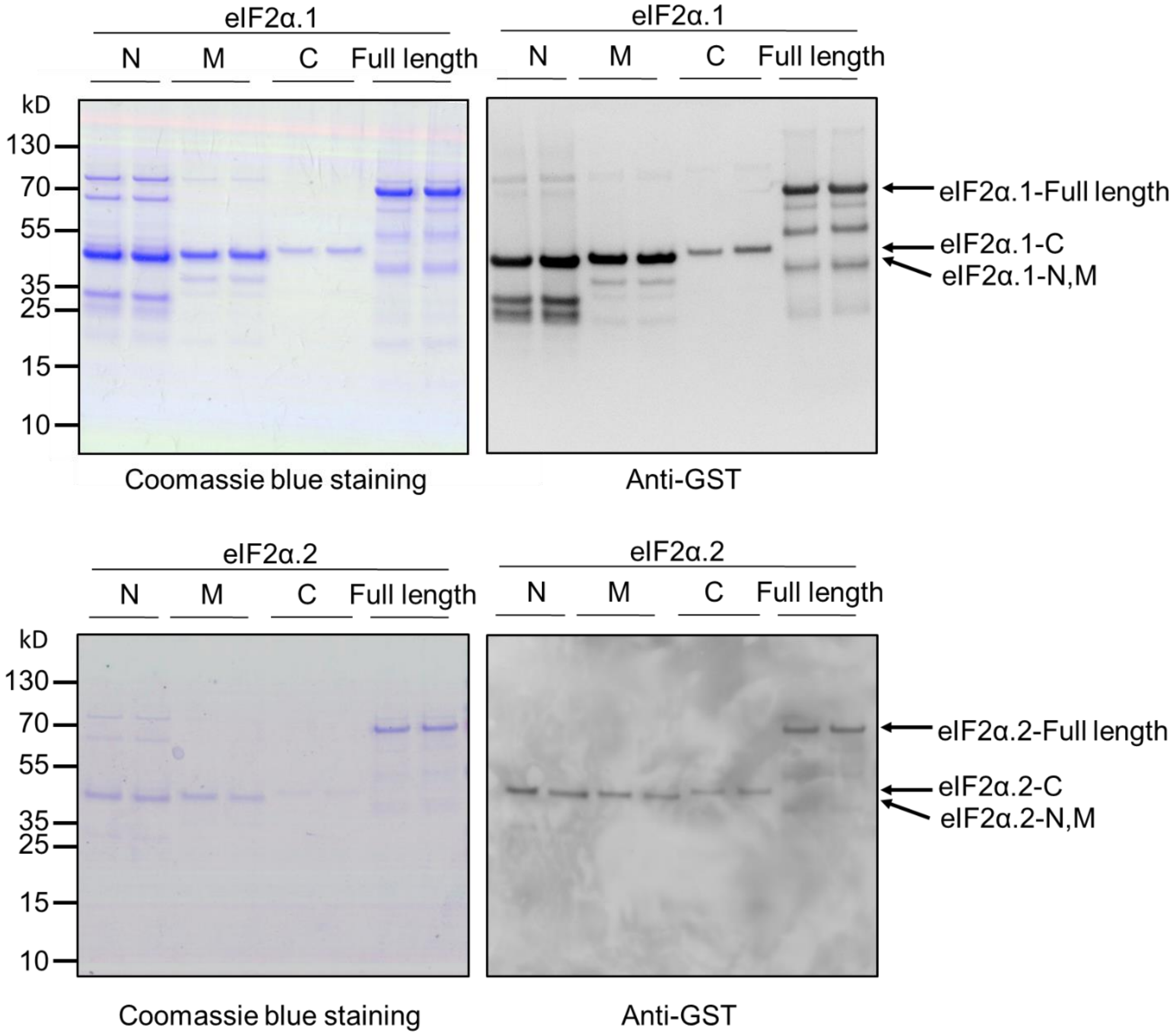


**Supplementary Figure 8: The protein amount of full-length and truncated fragments of eIF2*α*.2 detected in Coomassie blue staining gel and western blotting.**

The Coomassie blue staining gel (left) and western blotting (right) showed the equal amount of full-length and truncated fragments of eIF2α protein added in the kinase assay shown Fig. 4b, c. The proteins were examined by immunoblotting using antibody against GST tag. N, N-terminal domain; M, middle domain; C, C-terminal domain.

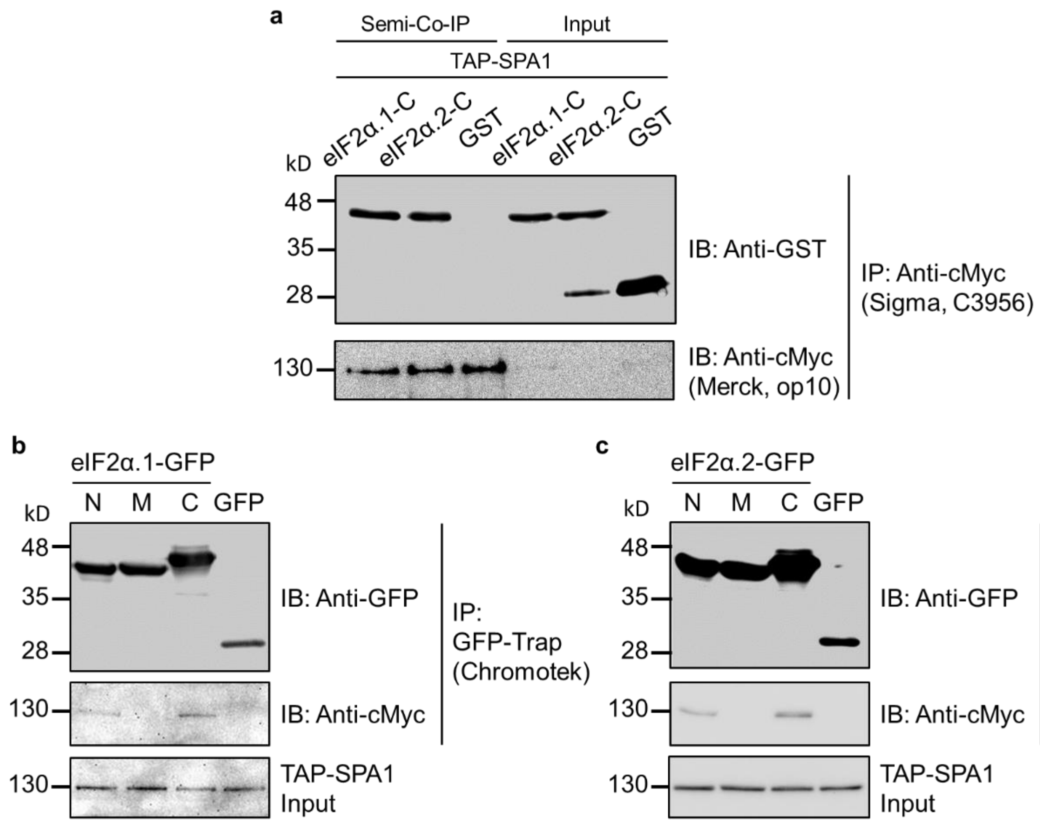


**Supplementary Figure 9: SPA1 interacts with the C-terminal domains of both eIF2α.1 and eIF2α.2 in the semi-*in vivo* and *in vivo* co-IP assay.**

**a** Semi-*in vivo* co-IP results showed that the C-terminal eIF2α.1/eIF2α.2 purified from *E. coli*. interacts with TAP-SPA1 extracted from Arabidopsis seedlings. The total proteins of 4-day-old TAP-SPA1 seedlings were incubated together with GST-tagged C-terminal eIF2α.1/eIF2α.2 (eIF2α.1-C/eIF2α.2-C) purified from *E. coli.* The mixture was incubated with Anti-cMyc (SIGMA, C3956). The precipitated proteins were examined by immunoblotting using antibodies against cMyc (MERCK, op10) and GST. GST only was used as negative control. Three biological repeats of data showed the same results.

**b, c** *In vivo* co-IP results showed that both N and C-terminal eIF2α.1/eIF2α.2-GFP interact with TAP-SPA1 in Arabidopsis seedlings. Five-day-old infected TAP-SPA1 etiolated seedlings were illuminated under 4 h light before sampling. The protein extract was incubated with GFP-Trap (Chromotek). The precipitated proteins were examined by immunoblotting using antibodies against GFP and cMyc. GFP only was used as negative control. N, N-terminal domain; M, middle domain; C, C-terminal domain. Three biological repeats of data showed the same results.

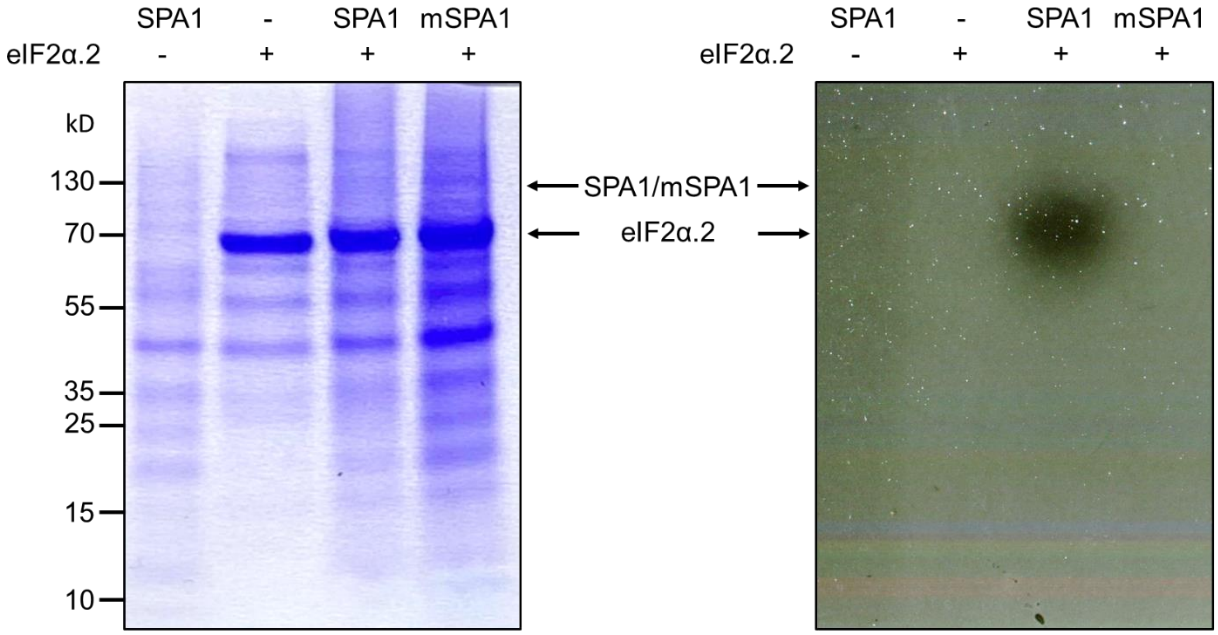


**Supplementary Figure 10: SPA1 phosphorylates eIF2α.2 *in vitro*.**

The *in vitro* kinase assay showed that SPA1 protein purified from *P. pastoris* phosphorylates the full-length of eIF2α.2 (autoradiogram on the right). A conserved amino acid mutation on the SPA1 kinase domain (mSPA1) reduced the phosphorylation activity of SPA1 on eIF2α. The left figure showed the protein levels in a Coomassie blue staining gel. -, no protein added; +, protein added.

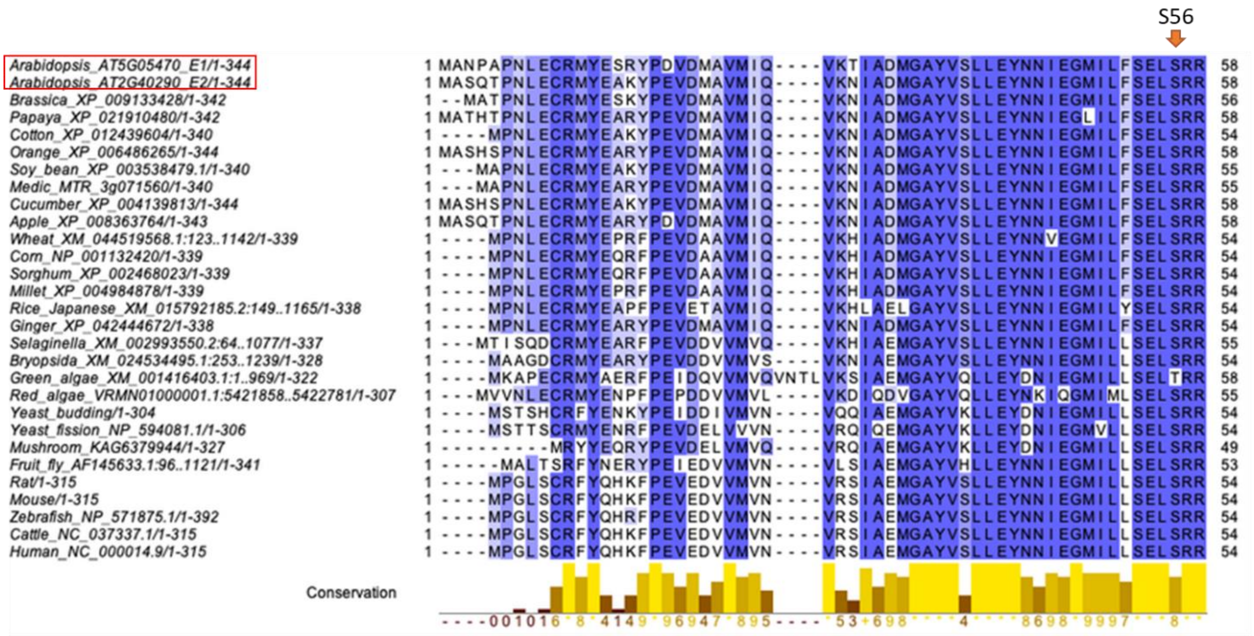


**Supplementary Figure 11: Alignment of amino acids surrounding the phosphorylation site at the N-terminal end of eIF2α showing conservation of the sequence in different species.** The species include angiosperm, gymnosperm, algae, yeast, fungus, insect and animals. The predicted conserved phosphorylation sites in different species are indicated by arrow. And the number of amino acids in Arabidopsis eIF2α.2 (AT2G40290) is labeled above the arrow.

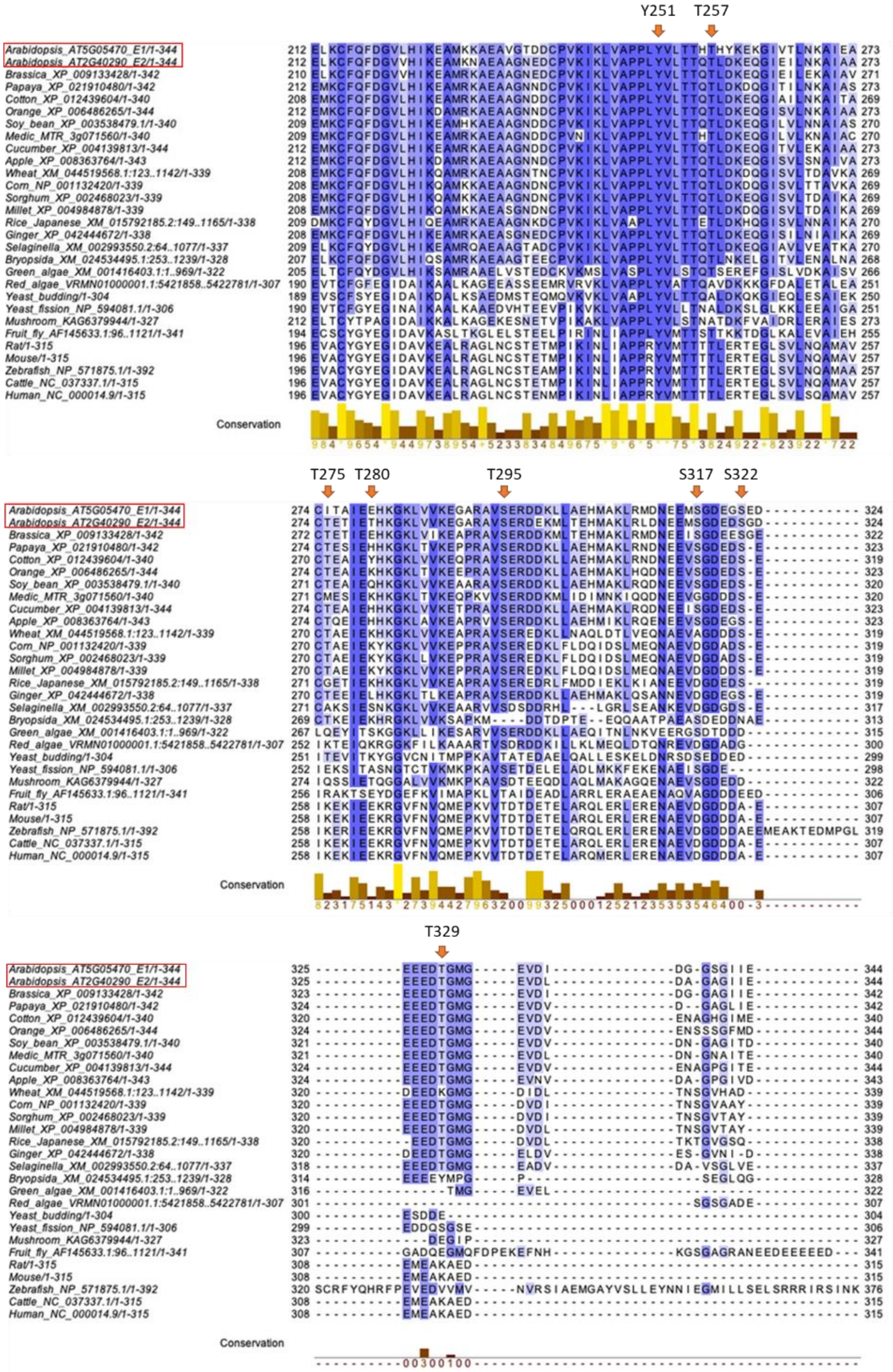

**Supplementary Figure 12: Alignment of amino acids surrounding the phosphorylation site at the C-terminal end of eIF2α showing conservation of the sequence in different species.** The species include angiosperm, gymnosperm, algae, yeast, fungus, insect and animals. The predicted conserved phosphorylation sites in different species are indicated by arrow. And the number of amino acids in Arabidopsis eIF2α.2 (AT2G40290) are labeled above the arrow.

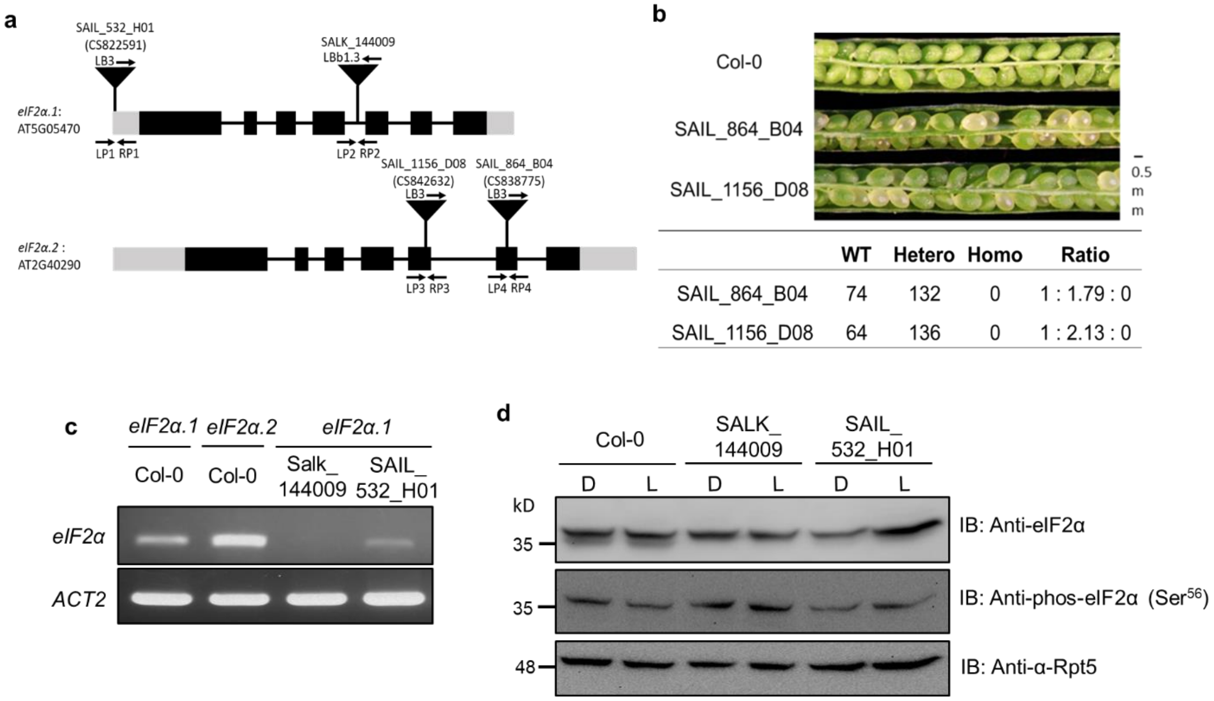


**Supplementary Figure 13: eIF2α.2 might act as a more important role compared with eIF2α.1.**

(a) Gene diagram of eIF2α.1 (AT5G05470) and eIF2α.2 (AT2G40290) to identify the T-DNA-inserted sites in *Arabidopsis thaliana* mutants. Exons and introns are represented by boxes (black, coding region; gray, UTR) and lines, respectively. Black arrowheads depict T-DNA insertions. Primers used for genotyping PCR and reverse transcription polymerase chain reaction (RT-PCR) are shown with arrows. (b) Seeds observation and genotyping results of eIF2α.2 T-DNA insertion mutants showed that *eif2α.2* mutants might be embryogenic lethal. Open eif2α +/- siliques showing white seeds among green seeds. (c) *eIF2α* mRNA expression in Col-0 and *eif2α.1* T-DNA insertion mutants. RT-PCR was performed to detect *eIF2α.1* and *eIF2α.2* expression in different mutant lines. *ACT2* was used as a internal control. (d) The total proteins of Col-0 and *eif2α.1* mutants with two biological repeats were loaded and separated on SDS-PAGE gel. The protein of eIF2α homologs were examined by immunoblotting using antibodies against eIF2α. Rpt5 protein was used as loading control.

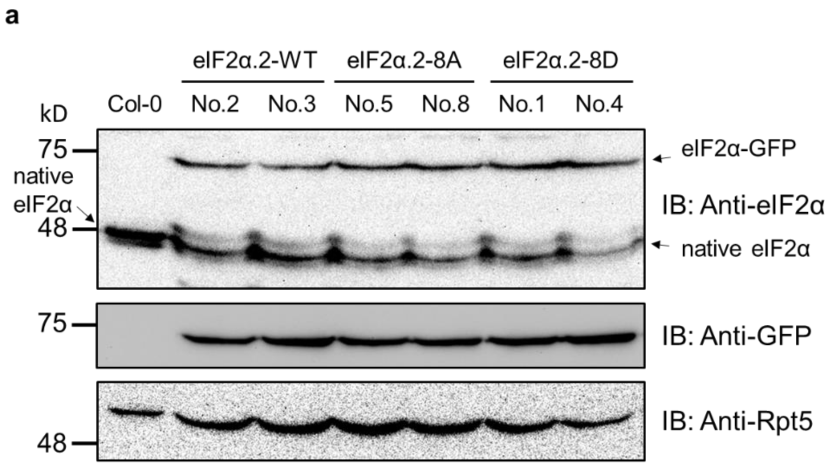


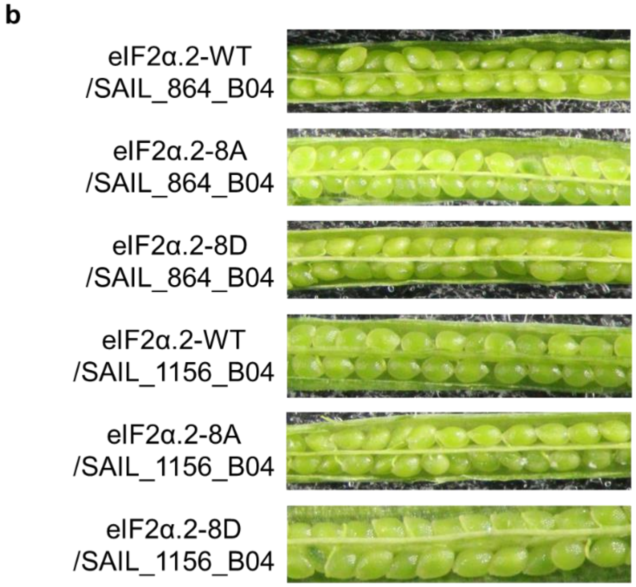


**Supplementary Figure 14: The native eIF2α protein expressions of eIF2α.2-GFP transgenic plants and seed development in eIF2α.2-GFP complementary lines.**

**a** The total proteins of 4-day-old Col-0 and eIF2α.2-GFP transgenic plants were loaded and separated on SDS-PAGE gel. The native and transgenic eIF2α protein levels were examined by immunoblotting using antibodies against eIF2α and GFP. Rpt5 protein was used as loading control.

**b** Opened siliques showing green seeds without aborted white seeds. Seeds observation of eIF2α.2-GFP variants transgenic plants in T-DNA inserted eIF2α.2 homozygous mutants (SAIL_864 and SAIL_1156) showed that eIF2α.2-GFP expressed in *eif2α.2* homozygous mutants could rescue the embryogenic lethal phenotype.

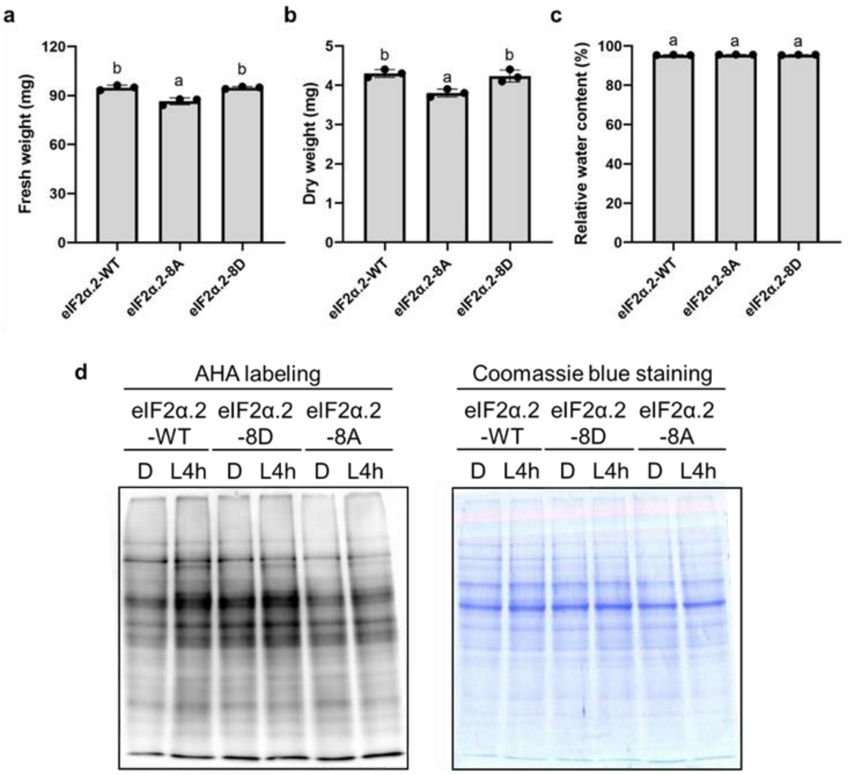


| | eIF2α.2-WT | eIF2α.2-8D | eIF2α.2-8A |
|---|---|---|---|
| Dark (D) | 1.00 | 1.32 ± 0.21 | 1.02 ± 0.09 |
| Light (L) | 1.25 ± 0.17 | 1.40 ± 0.14 | 1.01 ± 0.06 |

**Supplementary Figure 15: Phosphorylated eIF2α.2 promotes protein production as shown by measurements of fresh, dry weight, and *de novo* protein synthesis.**

**a, b, c** Bar graphs showing the fresh weight (a), dry weight (b) and relative water content (c) of 4-day-old etiolated seedlings of eIF2α.2-WT, eIF2α.2-8A, and eIF2α.2-8D transgenic lines. Error bars indicate the mean ± SD (n = 3). *$p$-value < 0.01, two-sided student's t-test.

**d** The figures showed *de novo* protein synthesis (left) examined by immunoblotting using antibodies against streptavidin, and the total protein content (right) stained with Coomassie blue. Four-day-old etiolated seedlings of eIF2α.2-WT, eIF2α.2-8A, and eIF2α.2-8D transgenic lines were incubated either under dark (D) or illuminated under white light for 4 h (L4h) before sampling. The table below the figures showed the relative signal intensities of *de novo* protein synthesis normalized to total protein content. The ratio of the dark condition of eIF2α.2-WT was set to 1 for analysis. Data of signal intensities are mean ± SD from three biological replicates.

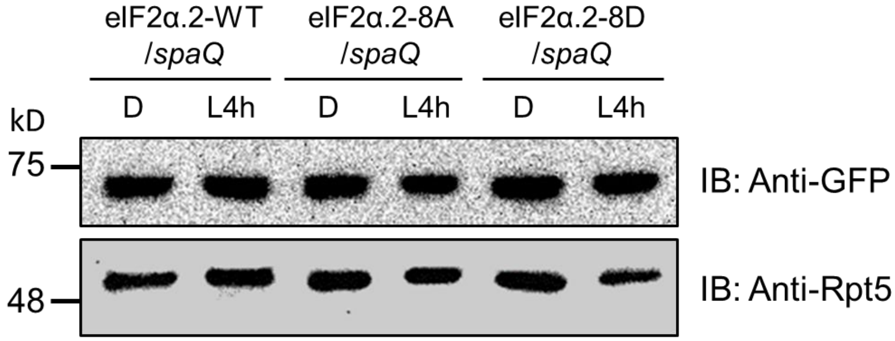


**Supplementary Figure 16: Detection of eIF2α.2-GFP variant protein transiently expressed in *spaQ* mutant background.**

Five-day-old etiolated *spaQ* seedlings transfected using AGROBEST system to transiently express the eIF2α.2 variants were incubated either under dark (D) or illuminated with 4 h white light (L4h) before sampling. Total proteins were loaded and separated on SDS-PAGE gel. The transgenic eIF2α protein levels were examined by immunoblotting using antibodies against GFP. Rpt5 protein was used as loading control.

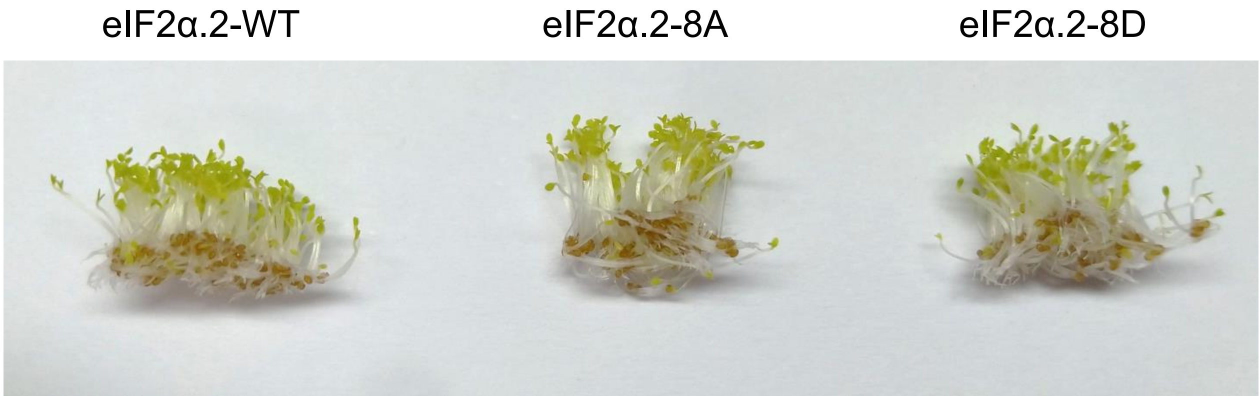


**Supplementary Figure 17: Phosphorylation of the C-terminal domain of eIF2α promotes the cotyledon greening.**

Photographs show the cotyledon greening of eIF2α.2-WT, eIF2α.2-8A, and eIF2α.2-8D transgenic lines. Etiolated seedlings of various genotypes were grown on MS medium under dark for 3 d and then illuminated with 4 h white light.

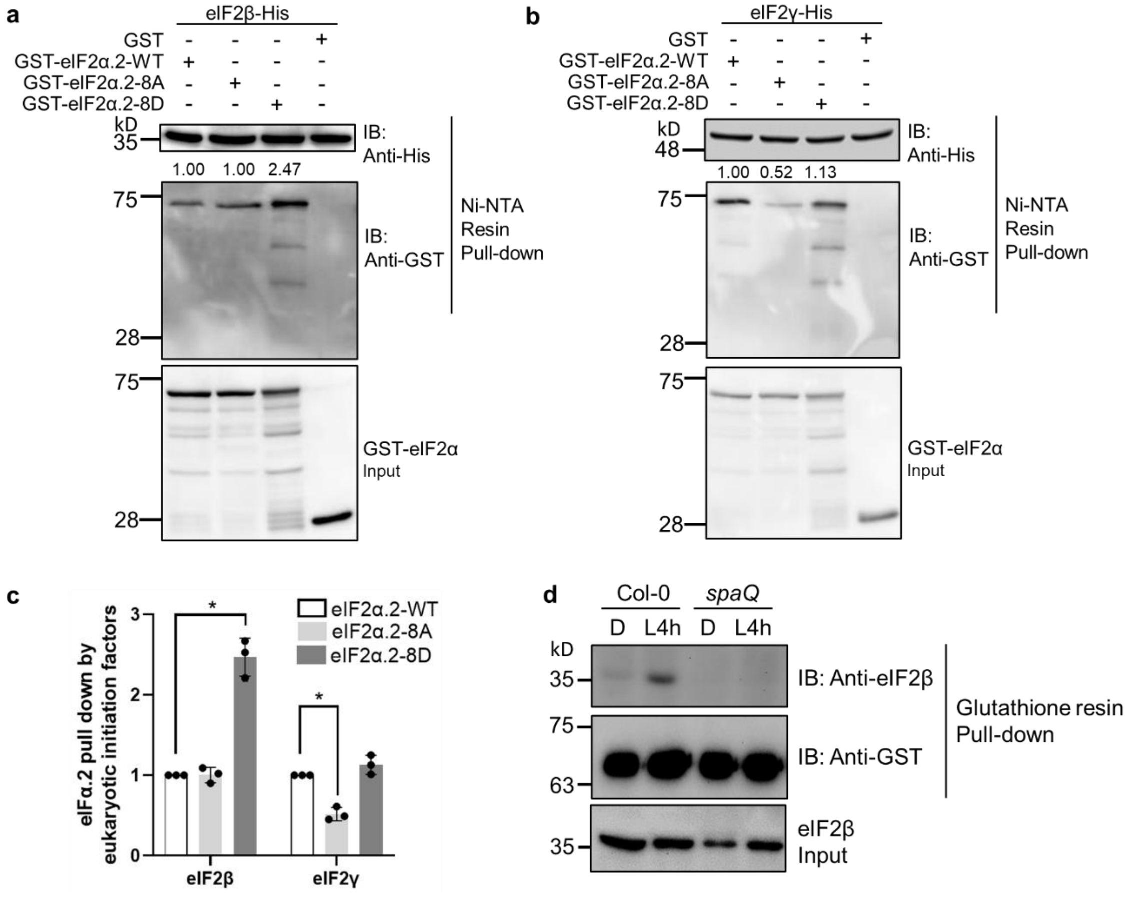


**Supplementary Figure 18: eIF2α.2 showed higher interaction intensity with eIF2β in Col-0 than in *spaQ* after light treatment as verified with semi-*in vivo* pull-down assay.**

**a, b** Pull-down assays showed eIF2β (a) and eIF2γ (b) have different interaction intensity with different phosphorylated forms of eIF2α.2 *in vitro*. The eIF2β, eIF2γ, and different mutative types of eIF2α.2 were expressed and purified from *E. coli.* The numbers below the upper panel indicate the relative band intensities of co-precipitated or pull-down protein normalized to those of precipitated protein. The ratio of the wild type (WT) of eIF2α group was set to 1 for analyzing the interaction intensities between the different mutative type of eIF2α. The tag proteins, GFP and GST, were used as a negative control.

**c** Bar graph showing the interaction between different mutative types of eIF2α.2 with eIF2β and eIF2γ *in vitro*. Error bars indicate the mean ± SD (n = 3). **p*-value < 0.01, two-sided student's t-test.

**d** Semi-*in vivo* pull-down assays showed that eIF2α.2 has higher interaction intensity with the native eIF2β in Col-0 after light treatment. The GST-eIF2α.2 (full-length) was expressed and purified from *E. coli*. The purified GST-eIF2α.2 were mixed with total proteins of 4-day-old Col-0 or *spaQ* seedlings incubated either under dark (D) or illuminated with 4 h white light (L4h) before sampling. The mixture was incubated with Glutathione resin. The pulled proteins were examined by immunoblotting using antibodies against eIF2β (kindly provided by Dr. Karen Browning) and GST. The lowest panel shows the native eIF2β detected by antibody in the total protein of Col-0 and *spaQ* seedlings before incubation.

**Supplementary Table 1: Primers used in this study**

| Characterization of transgene (RT-PCR) | |
|---|---|
| Primer | Sequence (5' to 3') |
| SALK_144009-LP | TAACCATGTTTGGGGTCAAAC |
| SALK_144009-RP | TCCTGATTCAGTTTTCGATGC |
| SAIL_532_H01-LP | AAGCATGAATCTTCGTGTTCG |
| SAIL_532_H01-RP | TACCTCCAAATCGACACCAAC |
| SAIL_864_B04-LP | CTCGGAGGTTTTTCTACTGCC |
| SAIL_864_B04-RP | CCTGCTGTTACGGAAGAAGTG |
| SAIL_1156_D08-LP | ATCTTACTCGATGATTCCGGC |
| SAIL_1156_D08-RP | ACGGATCCTGATTCAGTGTTG |
| LB3 | TAGCATCTGAATTTCATAACCAATCTCGATACAC |
| LBb1.3 | ATTTTGCCGATTTCGGAAC |
| PP2A-F | TATCGGATGACGATTCTTCGTGCAG |
| PP2A-R | GCTTGGTCGACTATCGGAATGAGAG |
| M13 Forward | GTA AAA CGA CGG CCA G |
| M13 Reverse | CAG GAA ACA GCT ATG AC |
| pEarleyGate 103-F | CGTGGAAAAAGAAGACGTTCCAACCAC |
| pEarleyGate 103-R | GTGCCGCTTCATATGATCTGGGTATCTT |
| eIF2α.1-F | ATGGCGAATCCTGCTCCG |
| eIF2α.1-R | TTCAATTATCCCGCTACCTCC |
| eIF2α.2-F | ATGGCGAGTCAAACACCGAATC |
| eIF2α.2-R | CTCGATGATTCCGGCACC |

| Cloning | |
|---|---|
| Primer | Sequence (5' to 3') |
| SPA1 (N545/C696/FL)<br>-SmaI-F | TCCCCCGGGAATGCCTGTTATGGAAAGAGT |
| SPA1 (C509/CC)-SmaI-F | TCCCCCGGGAGATATACTAAAGTCAGAGTTG |
| SPA1 (WD40)-SmaI-F | TCCCCCGGGACGGTATAGCAAGTTCGAAA |

| | |
|---|---|
| SPA1 (C509/WD40/FL)-SalI-R | CGCGTCGACTCAAACAAGTTTTAGTAGCTT |
| SPA1 (CC/C696)-SalI-R | CGCGTCGACGTGTTCTTCATCAGGGTCAAA |
| SPA1 (N545)-SalI-R | CGCGTCGACAGATATCTCCTCAGCAGCAG |
| eIF2α.2-Y251A-F | GTTGCTCCACCTCTGGCTG CCTTACTACTCA |
| eIF2α.2-Y251A-R | TGAGTAGTAAGGACAGCCAGAGGTGGAGCAAC |
| eIF2α.2-T275A-F | GCCATAGCAGCATGCGCTGAGACAATTGAGA |
| eIF2α.2-T275A-R | TCTCAATTGTCTCAGCGCATGCTGCTATGGC |
| eIF2α.2-T257A-F | GTCCTTACTACTCAGGCACTTGACAAGGAAC |
| eIF2α.2-T257A-R | GTTCCTTGTCAAGTGCCTGAGTAGTAAGGAC |
| eIF2α.2-T280A-F | GCTGAGACAATTGAGGCACACAAAGGCAAGC |
| eIF2α.2-T280A-R | GCTTGCCTTTGTGTGCCTCAATTGTCTCAGC |
| eIF2α.2-S295A-F | GAGGGGGCTAGAGCTGTGGCTGAACGTGATGAAAAGAT |
| eIF2α.2-S295A-R | ATCTTTTCATCACGTTCAGCCACAGCTCTAGCCCCCTC |
| eIF2α.2-S317A/S322A-F | TGAAGAAATGGCCGGCGATGAAGATGCCGGAGACGAA |
| eIF2α.2-S317A/S322A-R | TTCGTCTCCGGCATCTTCATCGCCGGCCATTTCTTCA |
| eIF2α.2-T329A-F | GACGAAGAAGAGGACGCTGGTATGGGCGAAG |
| eIF2α.2-T329A-R | CTTCGCCCATACCAGCGTCCTCTTCTTCGTC |
| eIF2α.2-Y251D/T257D-F | TCCACCTCTGGATGTCCTTACTACTCAGGACCTTGACAAGG |
| eIF2α.2-Y251D/T257D-R | CCTTGTCAAGGTCCTGAGTAGTAAGGACATCCAGAGGTGGA |
| eIF2α.2-T275D/T280D-F | AGCAGCATGCGATGAGACAATTGAGGATCACAAAGGCA |
| eIF2α.2-T275D/T280D-R | TGCCTTTGTGATCCTCAATTGTCTCATCGCATGCTGCT |
| eIF2α.2-S295D-F | GGGGCTAGAGCTGTGGATGAACGTGATGAAAA |
| eIF2α.2-S295D-R | TTTTCATCACGTTCATCCACAGCTCTAGCCCC |
| eIF2α.2-S317D/S322D-F | TGAAGAAATGGACGGCGATGAAGATGACGGAGACGAA |
| eIF2α.2-S317D/S322D-R | TTCGTCTCCGTCATCTTCATCGCCGTCCATTTCTTCA |
| eIF2α.2-T329D-F | GACGAAGAAGAGGACGATGGTATGGGCGAAGT |
| eIF2α.2-T329D-R | ACTTCGCCCATACCATCGTCCTCTTCTTCGTC |

## Supplementary Table 2: List of antibodies

| Target | Manufacturer | Catalogue No. | Dilution |
|---|---|---|---|
| **eIF2α** | Gifts from Dr. Karen S. Browning | | WB: 1:1000 in TBST |
| **eIF2β** | Gifts from Dr. Karen S. Browning | | WB: 1:1000 in TBST |
| **eIF2α-P51** | Cell Signaling | 9721 | WB: 1:1000 in TBST |
| **cMyc** | Sigma | C3956 | IP: 2.5ug per sample |
| **cMyc** | MERCK | OP10 | WB: 1:1000 in TBST |
| **GFP** | Abcam | ab6556 | IP: 2.5ug per sample |
| **GFP** | Santa Cruz | Sc-9996 | WB: 1:500 in TBST |
| **MBP** | NEB | E8032S | WB: 1:10000 in TBST |
| **Strep-HRP** | Thermo Scientific | N100 | WB: 1:4000 in TBST |
| **GST-HRP** | GE healthcare | RPN1236 | WB: 1:10000 in TBST |
| **RPT5** | ENZO lifescience | BML-PW8770-0025 | WB: 1:5000 in TBST |
| **His** | Bioman | HIT-001M | WB: 1:5000 in TBST |
| **Histon-H3** | Abcam | ab1791 | WB: 1:5000 in TBST |
| **Tubulin** | Sigma | T5168 | WB: 1:5000 in TBST |
