# Supplementary material for: The phosphorylation of carboxyl-terminal eIF2α by SPA kinases contributes to enhanced translation efficiency during photomorphogenesis
Source: Nat Commun. 2024 Apr 24;15:3467. doi: 10.1038/s41467-024-47848-7 (PMC11043401; doi:10.1038/s41467-024-47848-7)
Supplement: Supplementary file 3 — Description of Additional Supplementary Files [file 41467_2024_47848_MOESM3_ESM.pdf]

Description of Additional Supplementary Files

File Name: Supplementary Data 1

Description: List of SPA regulated genes which are regulated under both dark and light condition in Cluster 1.

File Name: Supplementary Data 2

Description: List of SPA regulated genes which are regulated under both dark and light condition in Cluster 2.

File Name: Supplementary Data 3

Description: List of SPA regulated genes which are regulated under both dark and light condition in Cluster 3.

File Name: Supplementary Data 4

Description: List of SPA regulated genes which are regulated under both dark and light condition in Cluster 4.

File Name: Supplementary Data 5

Description: GO analysis of SPA1 regulated genes on both dark and light condition in Cluster 1.

File Name: Supplementary Data 6

Description: GO analysis of SPA1 regulated genes on both dark and light condition in Cluster 2.

File Name: Supplementary Data 7

Description: GO analysis of SPA1 regulated genes on both dark and light condition in Cluster 3.

File Name: Supplementary Data 8

Description: GO analysis of SPA1 regulated genes on both dark and light condition in Cluster 4.
