# Supplementary material for: The phosphorylation of carboxyl-terminal eIF2α by SPA kinases contributes to enhanced translation efficiency during photomorphogenesis
Source: Nat Commun. 2024 Apr 24;15:3467. doi: 10.1038/s41467-024-47848-7 (PMC11043401; doi:10.1038/s41467-024-47848-7)
Supplement: Supplementary file 12 — Reporting Summary [file 41467_2024_47848_MOESM12_ESM.pdf]

nature portfolio

Corresponding author(s): Mei-Chun Cheng

Last updated by author(s): Mar 13, 2024

Reporting Summary

Nature Portfolio wishes to improve the reproducibility of the work that we publish. This form provides structure for consistency and transparency in reporting. For further information on Nature Portfolio policies, see our Editorial Policies and the Editorial Policy Checklist.

Statistics

For all statistical analyses, confirm that the following items are present in the figure legend, table legend, main text, or Methods section.

n/a	Confirmed	
☐	☒	The exact sample size (n) for each experimental group/condition, given as a discrete number and unit of measurement
☐	☒	A statement on whether measurements were taken from distinct samples or whether the same sample was measured repeatedly
☐	☒	The statistical test(s) used AND whether they are one- or two-sided<br>*Only common tests should be described solely by name; describe more complex techniques in the Methods section.*
☒	☐	A description of all covariates tested
☒	☐	A description of any assumptions or corrections, such as tests of normality and adjustment for multiple comparisons
☐	☒	A full description of the statistical parameters including central tendency (e.g. means) or other basic estimates (e.g. regression coefficient) AND variation (e.g. standard deviation) or associated estimates of uncertainty (e.g. confidence intervals)
☐	☒	For null hypothesis testing, the test statistic (e.g. F, t, r) with confidence intervals, effect sizes, degrees of freedom and P value noted<br>*Give P values as exact values whenever suitable.*
☒	☐	For Bayesian analysis, information on the choice of priors and Markov chain Monte Carlo settings
☒	☐	For hierarchical and complex designs, identification of the appropriate level for tests and full reporting of outcomes
☒	☐	Estimates of effect sizes (e.g. Cohen's d, Pearson's r), indicating how they were calculated

Our web collection on statistics for biologists contains articles on many of the points above.

Software and code

Policy information about availability of computer code

Data collection	No software was used for data collection.
Data analysis	Following softwares were used to analyze RNA-seq data. Bowtie2 (v2.1.0), FeatureCounts (subread v2.0.1), StringTie (StringTir v2.1.4), DEseq (DEseq v1.39.0), bcl2fastp (Illumina) (v2.20), R www.r-project.org. For hypocotyl elongation, cotyledon opening measurements, and quantification of Western blot signals, imageJ NIH http://rsb.info.nih.gov/ij/ was used.

For manuscripts utilizing custom algorithms or software that are central to the research but not yet described in published literature, software must be made available to editors and reviewers. We strongly encourage code deposition in a community repository (e.g. GitHub). See the Nature Portfolio guidelines for submitting code & software for further information.

Data

Policy information about availability of data

All manuscripts must include a data availability statement. This statement should provide the following information, where applicable:

- Accession codes, unique identifiers, or web links for publicly available datasets
- A description of any restrictions on data availability
- For clinical datasets or third party data, please ensure that the statement adheres to our policy

The authors declare that all data in this study are available in the figures and accompanying Supplementary Information file. RNA-seq raw data have been deposited

to the Gene Expression Omnibus database. Accession number: GSE233765. Arabidopsis mutants and transgenic lines, as well as plasmids and antibodies generated in this study are available from the corresponding author upon reasonable requests.

Research involving human participants, their data, or biological material

Policy information about studies with human participants or human data. See also policy information about sex, gender (identity/presentation), and sexual orientation and race, ethnicity and racism.

Reporting on sex and gender	N/A
Reporting on race, ethnicity, or other socially relevant groupings	N/A
Population characteristics	N/A
Recruitment	N/A
Ethics oversight	N/A

Note that full information on the approval of the study protocol must also be provided in the manuscript.

Field-specific reporting

Please select the one below that is the best fit for your research. If you are not sure, read the appropriate sections before making your selection.

☒ Life sciences ☐ Behavioural & social sciences ☐ Ecological, evolutionary & environmental sciences

For a reference copy of the document with all sections, see nature.com/documents/nr-reporting-summary-flat.pdf

Life sciences study design

All studies must disclose on these points even when the disclosure is negative.

Sample size	Three independently harvested and dried Arabidopsis thaliana seeds were used for phenotypic analysis. Each experiment represents results from at least 30 seedlings as indicated in the method section.
Data exclusions	No data exclusions.
Replication	All experiments were replicated at least three times.
Randomization	Experimental samples were numbered and experiments were performed by investigators.
Blinding	Investigators were blinded to group allocation.

Reporting for specific materials, systems and methods

We require information from authors about some types of materials, experimental systems and methods used in many studies. Here, indicate whether each material, system or method listed is relevant to your study. If you are not sure if a list item applies to your research, read the appropriate section before selecting a response.

Materials & experimental systems

n/a	Involved in the study	
☐	☒	Antibodies
☒	☐	Eukaryotic cell lines
☒	☐	Palaeontology and archaeology
☒	☐	Animals and other organisms
☒	☐	Clinical data
☒	☐	Dual use research of concern
☐	☒	Plants

Methods

n/a	Involved in the study	
☒	☐	ChIP-seq
☒	☐	Flow cytometry
☒	☐	MRI-based neuroimaging

Antibodies

Antibodies used	anti-eIF2α and anti-eIF2β (Gifts from Dr. Karen S. Browning, University of Texas at Austin), anti-eIF2α-P51 (Cell Signaling 9721), anti-

Antibodies used	cMyc (Sigma, C3956), anti-cMyc (MERCK OP10), anti-GFP (Abcam, ab6556), anti-GFP (Santa Cruz Sc-9996), anti-MBP (NEB E8032S), anti-strep-HRP (Thermo Scientific N100), anti-GST-HRP (GE healthcare RPN1236), anti-RPT5 (Enzo lifesciences BML-PW8770-0025), anti-His (Bioman HIT-001M), anti-Histon-H3 (Abcam ab1791), anti-Tubulin (Sigma T5168)
Validation	Antibodies are either previously validated (references indicated) or commercially available from companies as indicated.

Dual use research of concern

Policy information about dual use research of concern

Hazards

Could the accidental, deliberate or reckless misuse of agents or technologies generated in the work, or the application of information presented in the manuscript, pose a threat to:

No	Yes	
☒	☐	Public health
☒	☐	National security
☒	☐	Crops and/or livestock
☒	☐	Ecosystems
☒	☐	Any other significant area

Experiments of concern

Does the work involve any of these experiments of concern:

No	Yes	
☒	☐	Demonstrate how to render a vaccine ineffective
☒	☐	Confer resistance to therapeutically useful antibiotics or antiviral agents
☒	☐	Enhance the virulence of a pathogen or render a nonpathogen virulent
☒	☐	Increase transmissibility of a pathogen
☒	☐	Alter the host range of a pathogen
☒	☐	Enable evasion of diagnostic/detection modalities
☒	☐	Enable the weaponization of a biological agent or toxin
☒	☐	Any other potentially harmful combination of experiments and agents
